# Relationship between tumour shrinkage and reduction in Ki67 expression after primary chemotherapy in human breast cancer

**DOI:** 10.1054/bjoc.2001.2048

**Published:** 2001-10

**Authors:** A Bottini, A Berruti, A Bersiga, M P Brizzi, P Bruzzi, S Aguggini, A Brunelli, G Bolsi, G Allevi, D Generali, E Betri, G Bertoli, P Alquati, L Dogliotti

**Affiliations:** Centro di Senologia, Servizio di Anatomia Patologica, Azienda Ospedaliera Istituti Ospitalieri, Viale Concordia 1, Cremona, 26100; Oncologia Medica, Dipartimento di Scienze Cliniche e Biologiche, Università di Torino, Azienda Ospedaliera San Luigi, Regione Gonzole 10, Orbassano, 10043, Torino,; Istituto Nazionale per la Ricerca sul Cancro, Largo R. Benzi 10, Genova, 16132, Italy

**Keywords:** Ki67, epirubicin, CMF, tamoxifen

## Abstract

The association between tumour shrinkage and reduction in kinetic cell activity after primary chemotherapy in human breast cancer is still a matter of investigation. 157 patients with T2-4, N0-1, M0 breast cancer received primary chemotherapy consisting of either the CMF regimen + tamoxifen (the first consecutive 76 cases) or the single agent epirubicin (the subsequent 81). Ki67, p53, bcl2, c-erbB2 and steroid hormone receptors were evaluated immunohistochemically in tumour specimens obtained before chemotherapy and at surgery. Tumour shrinkage of >50% occurred in 72.4% of patients. Ki67 expression significantly decreased after chemotherapy; the reduction correlated with tumour response in both univariate (*P* < 0.005) and multivariate analysis (*P* = 0.02). p53, bcl-2, steroid hormone receptor and c-erbB2 immunostaining were scarcely affected. Baseline bcl2 (*P* = 0.04) and c-erbB2 (*P* = 0.02) were directly and inversely associated with the reduction in Ki67 immunostaining, respectively. Baseline p53 expression (*P* < 0.01) was directly related with Ki67 expression at residual tumour, whereas oestrogen receptor expression (*P* < 0.001) was inversely related. Ki67 at residual tumour was a better predictor for relapse-free survival (RFS) than baseline Ki67. Clinical response (*P* < 0.03), but not reduction in Ki67, was a significant independent predictor for disease recurrence. Chemotherapy was found to induce tumour shrinkage and to reduce the number of cells in the cell cycle, but its effect on tumour biology/aggressiveness was minimal. Reduction in Ki67 immunostaining correlated with clinical response but failed to be related to RFS. Ki67 expression at surgery rather than at baseline appears to be a better predictor for disease relapse. © 2001 Cancer Research Campaign  http://www.bjcancer.com


*British Journal of Cancer* (2001) **85**(8), 1106–1112
© 2001 Cancer Research Campaign
doi: 10.1054/ bjoc.2001.2048, available online at http://www.idealibrary.com on IDEAL

http://www.bjcancer.com

### Relationship between tumour shrinkage and reduction in Ki67 expression after primary chemotherapy in human breast cancer

**A Bottini[1], A Berruti[3], A Bersiga[2], MP Brizzi[3], P Bruzzi[4], S Aguggini[1], A Brunelli[1], G Bolsi[2], G Allevi[1], D Generali[1], E Betri[2], G Bertoli[2], P Alquati[1] and L Dogliotti[3]**

[1]Centro di Senologia and [2]Servizio di Anatomia Patologica, Azienda Ospedaliera Istituti Ospitalieri, Viale Concordia 1, 26100 Cremona; [3]Oncologia Medica, Dipartimento di Scienze Cliniche e Biologiche, Università di Torino, Azienda Ospedaliera San Luigi, Regione Gonzole 10, 10043 Orbassano, Torino, [4]Istituto Nazionale per la Ricerca sul Cancro, Largo R. Benzi 10, 16132 Genova, Italy

**Summary** The association between tumour shrinkage and reduction in kinetic cell activity after primary chemotherapy in human breast cancer is still a matter of investigation. 157 patients with T2-4, N0-1, M0 breast cancer received primary chemotherapy consisting of either the CMF regimen + tamoxifen (the first consecutive 76 cases) or the single agent epirubicin (the subsequent 81). Ki67, p53, bcl2, c-erbB2 and steroid hormone receptors were evaluated immunohistochemically in tumour specimens obtained before chemotherapy and at surgery. Tumour shrinkage of >50% occurred in 72.4% of patients. Ki67 expression significantly decreased after chemotherapy; the reduction correlated with tumour response in both univariate ($P < 0.005$) and multivariate analysis ($P = 0.02$). p53, bcl-2, steroid hormone receptor and c-erbB2 immunostaining were scarcely affected. Baseline bcl2 ($P = 0.04$) and c-erbB2 ($P = 0.02$) were directly and inversely associated with the reduction in Ki67 immunostaining, respectively. Baseline p53 expression ($P < 0.01$) was directly related with Ki67 expression at residual tumour, whereas oestrogen receptor expression ($P < 0.001$) was inversely related. Ki67 at residual tumour was a better predictor for relapse-free survival (RFS) than baseline Ki67. Clinical response ($P < 0.03$), but not reduction in Ki67, was a significant independent predictor for disease recurrence. Chemotherapy was found to induce tumour shrinkage and to reduce the number of cells in the cell cycle, but its effect on tumour biology/aggressiveness was minimal. Reduction in Ki67 immunostaining correlated with clinical response but failed to be related to RFS. Ki67 expression at surgery rather than at baseline appears to be a better predictor for disease relapse. © 2001 Cancer Research Campaign http://www.bjcancer.com

**Keywords**: Ki67; epirubicin; CMF; tamoxifen

The administration of primary chemotherapy in breast cancer (BC) patients permits in vivo assessment of tumour chemosensitivity and evaluation of treatment-induced changes in tumour biology. Although administered according to different schedules, primary chemotherapy has repeatedly demonstrated it can be highly active in downstaging BC, thus facilitating subsequent conservative surgical approaches (Bonadonna, 1990; Mauriac et al, 1991; Powles et al, 1995; Fisher et al, 1997; Makris et al, 1998). In addition, several courses of primary chemotherapy have been reported to significantly inhibit tumour growth rate (Daidone et al, 1991; Gardin et al, 1994). In contrast, neither c-erbB2 nor steroid hormone receptor expression were consistently affected (Bottini et al, 2000; Frassoldati et al, 1997).

Accordingly, reduction in tumour size and in proliferative activity are so far considered the most relevant biological effects of chemotherapy on primary BC. However, an association between these 2 phenomena has not yet been determined.

In the present study, we evaluated the variation in the number of cells in the G1, S, G2 and M phases of the cell cycle by Ki67 immunostaining before and after chemotherapy in a series of BC patients diagnosed and followed up in the same institution. The primary aim of the study was to search for a relationship between the decline in Ki67 stained cells induced by the treatment and the clinical response. The secondary aims were: (1) to evaluate, either by univariate or multivariate analysis, the role of both response to treatment and Ki67 antigen expression (determined either at baseline or at the end of the treatment) in predicting the disease relapse; (2) to see whether changes in certain pre-treatment tumour characteristics, i.e. histology grade, steroid hormone receptor, c-erbB2, p53 and bcl2 expressions, before and after treatment, may be related to the tumour shrinkage.

*Received 6 December 2000*
*Revised 9 July 2001*
*Accepted 13 July 2001*

*Correspondence to:* L Dogliotti

### MATERIALS AND METHODS

### Patients

The study was carried out on 157 consecutive patients recruited in our institution from August 1990 to January 1997 with an operable breast tumour or locally advanced disease (T2-4N0-1M0). The patients had been enrolled in 2 consecutive phase II studies that aimed to evaluate the activity of the CMF regimen (cyclophosphamide, methotrexate, 5-fluorouracil) administered in association with tamoxifen in cases with oestrogen receptor positive (ER+) tumours, and that of the single agent epirubicin. None of the patients had objective skin inflammation or oedema. On first presentation an incision biopsy was performed on each patient. Initial staging comprised clinical examination, bilateral mammography, echography, chest X-ray, liver echography or CT scan, bone scintigraphy. All patients gave informed consent to the diagnostic procedures and the proposed treatment.

### Treatment

Chemotherapy was started within 1 or 2 days from diagnosis. The first consecutive 76 patients received the CMF (cyclophosphamide, methotrexate and 5-fluorouracil) chemotherapy regimen, which was given on days 1 and 8 every 28 days. The dose of cyclophosphamide and 5-fluorouracil was 600 mg $m^{-2}$ of body surface area, and the dose of methotrexate was 40 mg $m^{-2}$. The subsequent 81 patients received epirubicin 60 mg $m^{-2}$ on days 1 and 2 every 21 days. The first consecutive 45 patients, with oestrogen positive (ER+) BC at first biopsy, received additional tamoxifen (TAM) treatment (30 mg daily) in association with the CMF treatment. TAM was administered after obtaining the results of the receptor status, about 20 days from the first biopsy, and continued up until surgery. Each month the size of the primary tumour and the size of the axillary lymph-nodes, when appreciable, were carefully measured with a calliper by the same clinician. Response was assessed by the clinical measurement of the changes in the product of the two largest diameters recorded in two successive evaluations. According to the World Health Organization (WHO) criteria (World Health Organisation, 1978) tumour progression (PD) was defined as an increase of at least 25% in tumour size, stable disease (SD) as an increase of less than 25% or a reduction of less than 50%, partial response (PR) as a tumour shrinkage greater than 50%, and complete response (CR) as the complete disappearance of all clinical signs of disease.

Surgery was planned after full clinical reassessment. Quadrantectomy or modified radical mastectomy were performed when indicated in association with full axillary dissection. All patients subjected to quadrantectomy underwent irradiation of the residual breast (60 Gy delivered over 6 weeks).

### Histopathologic grade and immunohistochemistry

The degree of malignancy was assessed according to the Elston and Ellis grading system which classifies tumours into grade I (well differentiated), grade II (moderately differentiated), and grade III (poorly differentiated) (Elston and Ellis, 1991).

The immunohistochemical assays used in this study are fully described elsewhere (Bottini et al, 2000). Briefly, an antigen retrieval step was performed by heating a tissue section in a citrate buffer. The primary antibodies applied were: ER [mouse monoclonal 6F11 (Novocastra Lab, Newcastle upon Tyne, UK), dilution 1:50, 1 h incubation at RT]; PgR [mouse monoclonal 1A6 (Novocastra Lab), dilution 1:20, 1 h incubation at RT]; Ki67 [mouse monoclonal Mib-1 (Dako, Glostrup, Denmark), dilution 1:30, 1 h incubation at RT]; p53 [mouse monoclonal D07 (Novocastra Lab), dilution 1:100, 1 h incubation at RT]; bcl-2 [mouse monoclonal 124 (Dako), dilution 1:40, overnight at 4˚C]; c-erbB2 [mouse monoclonal CB11 (Novocastra Lab), overnight at 4˚C].

Biotinylated horse anti-mouse IgG and avidin–biotin–peroxidase complex were applied as a staining method (Vectastatin ABC kit; Vector Laboratories, Inc, Burlingame, CA). A solution containing hydrogen peroxide (0.06% v/v) and diamino-benzidine 4 HCL (DAB; 0.05 v/v) was used as chromogen.

### Immunohistochemical scoring

All samples had a negative control slide (no primary antibody) of an adjacent section to assess the degree of non-specific staining. Positive controls included breast carcinomas known to exhibit high levels of each marker.

All staining was scored by counting the number of positive stained cells and expressed as a percentage of the total tumour cells (at least 1000) counted across several representative fields of the section using a standard light microscope equipped with a $10 \times 10$ square graticule. Reproducibility of counting was assessed by a second investigator re-scoring 10 slides.

The relative intensity of ER and PgR staining was assessed in a semi-quantitative fashion as previously described by McCarty et al (1985), incorporating both the intensity and distribution of specific staining. A value (HSCORE) was derived from the sum of the percentages of positive-stained epithelial cells multiplied by the weighted intensity of staining. Specimens were deemed receptor positive if the HSCORE was greater than 100 (Robertson et al, 1992). For the other biological parameters, a cut-off of ≥ 5 positive cells was introduced to discriminate p53-positive and p53-negative primary malignancies, as previously reported (Silvestrini et al, 1993), while no cut-offs were introduced for c-erbB2 and bcl2 expression. The immunohistochemical evaluation at mastectomy was performed by the same pathologists who remained blinded to the disease response and the score assessed at first biopsy.

### Statistical analysis

Ki67 staining was analysed both as a continuous variable and after categorisation into 3 classes (≤ 10%, 11–29%, ≥ 30%).

Non-parametric statistical methods (Mann–Whitney test for unpaired data, Wilcoxon's matched-pairs signed-rank test for paired data, Spearman rho for simple correlation analysis) were used in the primary analyses of the data. Multiple group comparison for Ki67 expression at baseline was performed by ANOVA. In order to take into account a possible confounding effect of baseline Ki67, ANCOVA was performed instead of ANOVA for multiple group comparison when considering the reduction in Ki67 expression and Ki67 staining at residual tumours. Associations among the variables were evaluated by the $\chi^2$ test. Multivariate analysis was performed by multiple linear regression. As the variable residual Ki67 after chemotherapy was not normally distributed, a square root transformation of this variable was used in the multiple regression and ANCOVA analyses. Relapse-free survival (RFS) was calculated from diagnosis to the occurrence of relapse of disease. The Cox model was employed to perform multivariate survival analysis for the prediction of disease recurrence. Statistical analysis was performed on an IBM-compatible personal computer using the Statistica for Windows (1995) software package.

### RESULTS

Patient characteristics at diagnosis are shown in Table 1. Most patients (75%) had infiltrating ductal carcinoma; infiltrating lobular carcinoma and mixed forms (infiltrating ductal + lobular) were found in 19% and 6%, respectively.

Figure 1 shows how baseline Ki67 expression correlated directly with ER and bcl2 expression but negatively with both c-erbB2 and p53 immunostaining. G3 tumours had higher Ki67

© 2001 Cancer Research Campaign 

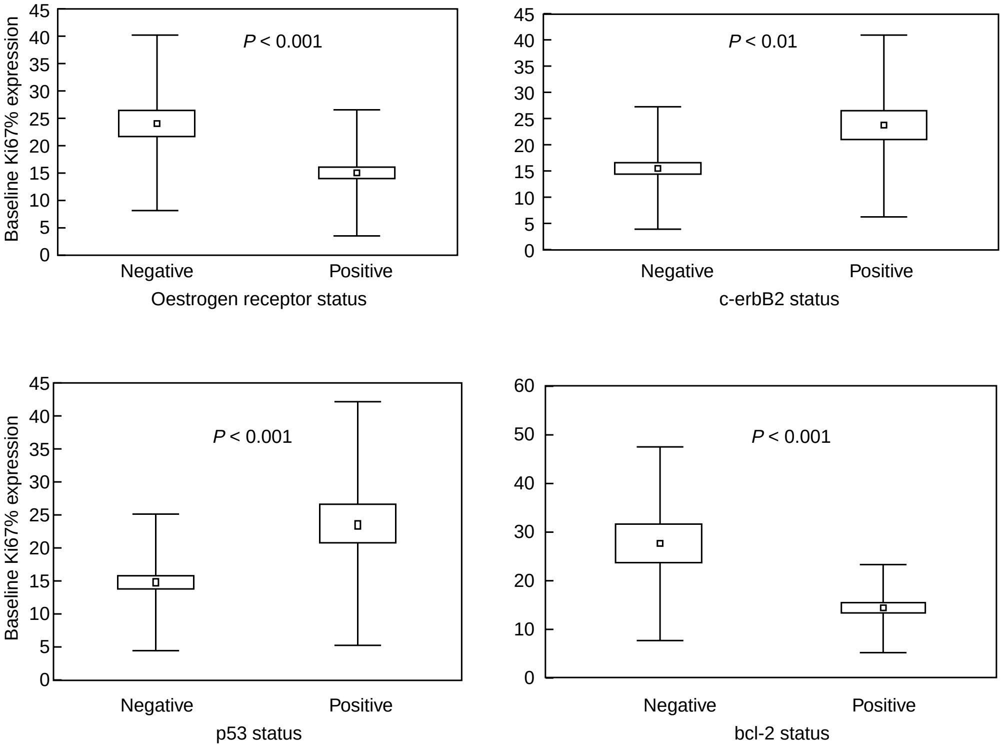

**Figure 1** Box and whisker plots of baseline distribution of Ki67% expression according to oestrogen receptors, c-erbB2, p53 and bcl-2 status. Data are: □ Mean; ▭ ± Standard error; ⌶ ± Standard deviation expression (median 16 (0–90)) than G2 (median 13 (1–55)), although the difference failed to attain statistical significance ($P < 0.07$). No significant association was observed between Ki67 expression and either tumour size (median 16 (0–50) and 16 (1–90) for T2 vs T3–4 tumours) or lymph node status (median 14.5 (1–50) and 16 (0–90) for N+ vs N– BC).

### Treatment activity

A median of 3 cycles was administered (range 3–5). 156 patients completed the chemotherapy treatment and were evaluable for response. One patient refused to continue the treatment plan at the end of the first cycle. 37 patients attained clinical CR (23.7%), 76 clinical PR (48.7%) for an overall response rate (CR + PR) of 72.4%, 40 patients showed SD (25.6%), while only 3 progressed (1.9%). Only 4 patients (2.6%) attained pathological complete remission, 2 of them with clinical CR and 2 with clinical PR.

According to the treatment administered, the distribution of CR, PR, SD and PD was 21/76 (27.6%), 39/76 (51.3%), 14/76 (18.4%) and 2/76 (2.6%) for the patients submitted to CMF + TAM and 16/80 (20.0%), 37/80 (46.2%), 26/80 (32.5%) and 1/80 (1.3%) for those receiving the single agent epirubicin.

No statistically significant differences in response rates were found according to the pre-treatment Ki67 expression: 39/51 (76.5%), 57/83 (68.7%), and 17/23 (73.9%) overall response rates were found in tumours with ≤10% Ki67 positive cells, between 11% and 29% and ≥30%, respectively ($P = 0.67$); while the corresponding complete response rates were 16/51 (31.3%), 16/83 (19.3%) and 5/23 (21.7%), respectively ($P = 0.28$). Responses were equally distributed in c-erbB2-positive and -negative tumours in both the CMF+TAM subgroup and in the epirubicin subset.

All patients received radical surgery. Modified mastectomy was performed in 68 cases (43.6%) and quadrantectomy followed by radiation therapy in 88 (56.4%).

### Effect of chemotherapy +/– endocrine therapy on Ki67 staining

A total of 152 BC had Ki67 assessed before and after chemotherapy Ki67 was not assessable at mastectomy in 4 tumour samples due to pathological complete remission. The median number of cells per tumour expressing the Ki67 antigen fell from 16% (range: 0–90%) at the initial biopsy to 8% (range: 0–80%) at mastectomy ($P < 0.001$). At the end of the course of chemotherapy, 101 residual tumours (65.6%) had <10% Ki67 positive cells, 43 (27.9%) between 10% and 29%, 10 (6.5%) ≥30%. A moderate but highly significant relationship was found between Ki67 values before chemotherapy and those evaluated afterwards (Spearman $r = 0.49$, $P < 0.001$).

Figure 2C shows that Ki67 reduction was greater in patients who attained a clinical response than in non-responders. Ki67 expression at baseline was not different between patients who subsequently did or did not respond to treatment (Figure. 2A), whereas Ki67 evaluated at residual tumours after chemotherapy was lower in patients with complete response than in those with partial response or no change (Figure. 2B).

No significant difference in reduction in Ki67 expression was found in patients receiving CMF + TAM (mean: –7.34 ± SD 10.8) vs those submitted to epirubicin (mean –6.61 ± SD 14.2).

 *© 2001 Cancer Research Campaign*

**Table 1** Patient characteristics

| | |
|---|---|
| No. | 157 |
| Age | |
| Median | 56 |
| Range | 26–71 |
| Menopausal status | |
| Premenopause | 54 (34.4%) |
| Postmenopause | 103 (65.6%) |
| TNM | |
| $T_2$ | 110 (70.1%) |
| $T_3$ | 33 (21%) |
| $T_4$ | 14 (8.9%) |
| $N_0$ | 83 (52.9%) |
| $N_1$ | 74 (47.1%) |
| Grade | |
| II | 54 (34.4%) |
| III | 103 (65.6%) |
| Receptor status | |
| ER +[a] | 114 (72.6%) |
| ER – | 43 (27.4%) |
| PgR +[a] | 64 (41.6%) |
| PgR – | 90 (58.4%) |
| N.E.[b] | 3 |
| Ki67 | |
| ≤ 10% | 51 (32.5%) |
| 11–29% | 83 (52.9%) |
| ≥ 30% | 23 (14.6%) |
| c-erbB2 | |
| Positive | 38 (26.2%) |
| Negative | 107 (73.8%) |
| N.E.[b] | 12 |
| P53 | |
| positive | 41 (28.7%) |
| negative | 102 (71.3%) |
| N.E.[b] | 14 |
| bcl-2 | |
| positive | 106 (75.2%) |
| negative | 35 (24.8%) |
| N.E.[b] | 16 |

[a]ER+ (HSCORE > 100); PgR+ (HSCORE > 100). [b]Not evaluable. A cut off of 5% stained cells was introduced for defining p53 positive tumours, whereas no cut off was introduced for c-erbB2 and bcl2 staining.

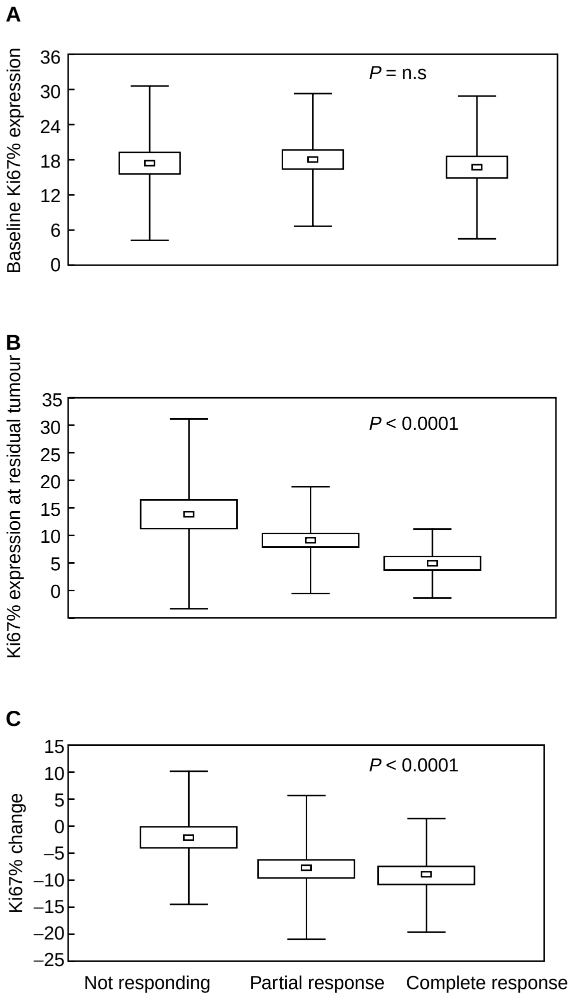

**Figure 2** Box and whisker plots of the distribution of Ki67% expression at baseline (**A**) and at residual tumour (**B**) to chemotherapy, according to the clinical response obtained. Reduction in Ki67 immunostaining according to the clinical response (**C**). Data are: ☐ Mean; ▭ ± Standard error; ⌶ ± Standard deviation

### Effect of cytotoxic treatment on histology grade, steroid hormone receptor status, p53, bcl2 and c-erbB2 expression

Oestrogen receptor expression at baseline decreased after chemotherapy in 57 specimens (37.8%), increased in 55 (36.4%), and remained unchanged in 39 (25.8%). The corresponding pattern for PgR was 52 (35.9%), 41 (28.2%), and 52 (35.9%), respectively. Histology grade was only minimally affected by the treatment, being unchanged in 138 specimens (93.2%), increased in 7 (4.8%) and decreased in 3 (2.0%). As regards p53 and bcl2 expression, it rose in 30 (21.4%) and 24 (17.5%) specimens after treatment, decreased in 17 (12.1%) and 20 (14.6%), and remained unchanged in 93 (66.5%) and 93 (67.9%), respectively. Finally, c-erbB2 immunostaining showed an increase from baseline in 18 specimens (12.9%), a decrease in 4 (2.9%) and no change in 117 (84.2%).

When these variables were considered as dichotomous (positive and negative), the changes after treatment were found to be even less evident. PgR-positive tumours before chemo-endocrine therapy administration became negative in 21.5% of cases, whereas the opposite pattern occurred in 15.2%. The other biological parameters did not change their baseline status in more than 10% of cases.

### Multivariate analysis of independent variables associated with clinical response

A multivariate logistic analysis was performed in order to search for clinical and biological parameters assessed at baseline as predictors for clinical response. Treatment, CMF + TAM vs epirubicin ($P = 0.005$), and postoperative pathologic lymph node status ($P = 0.05$) were the only variables associated with overall response. Tumour size, baseline clinical lymph node status, histology grade, KI67, c-erbB2, p53, bcl2, ER, PgR immunostaining and menopausal status did not enter the model.

When considering the changes in the biological parameters before and after treatment, reduction in Ki67 expression ($P = 0.02$) and treatment ($P = 0.05$) were independently associated with tumour shrinkage (complete + partial response), whereas the changes in c-erbB2, p53, bcl2 and steroid hormone receptor immunostaining did not enter the model (data not shown).

*© 2001 Cancer Research Campaign*

**Table 2** Independent variables associated with reduction in %Ki67 immunostaining or %Ki67 at residual tumours according to multivariate regression analysis

| **Ki67 reduction** | **B** | **SE B** | ***P*** |
|---|---|---|---|
| Tumour size[a] | 0.88 | 1.84 | 0.63 |
| Baseline lymph node status[a,b] | 0.04 | 2.27 | 0.98 |
| Post-chem lymph node status[a,c] | –1.00 | 2.38 | 0.67 |
| Histology grade[a] | 0.78 | 2.69 | 0.77 |
| c-erbB2 | –6.80 | 2.81 | <0.02 |
| p53[a] | 2.09 | 2.60 | 0.42 |
| bcl2 | 6.06 | 3.00 | <0.04 |
| ER[a] | 5.63 | 3.11 | 0.07 |
| PgR[a] | 1.62 | 2.67 | 0.54 |
| Menopause[a] | –0.85 | 2.48 | 0.73 |
| Treatment[a] (CMF + TAM vs epirubicin) | –2.27 | 2.32 | 0.33 |
| **Ki67 expression at residual tumours** | | | |
| Tumour size[a] | 1.00 | 1.50 | 0.50 |
| Baseline lymph node status[a,b] | 1.85 | 1.70 | 0.28 |
| Post-chem lymph node status[a,c] | –1.69 | 1.90 | 0.37 |
| Histology grade[a] | 0.92 | 2.15 | 0.67 |
| c-erbB2[a] | –0.23 | 2.30 | 0.92 |
| p53 | 5.02 | 2.09 | <0.02 |
| bcl2[a] | –4.72 | 2.46 | 0.06 |
| ER | –9.54 | 2.45 | <0.001 |
| PgR[a] | –0.80 | 2.19 | 0.72 |
| Menopause[a] | –0.04 | 2.01 | 0.98 |
| Treatment (CMF + TAM vs epirubicin) | 5.68 | 1.86 | <0.002 |

[a]Not included in the model. [b]Preoperative clinical lymph node status. [c]Post operative pathologic lymph node status. B = Estimated regression coefficient. S.E. = Standard Error of B. *P* = The significant level of the Wald statistics.

### Multivariate analysis of independent variables associated with reduction in Ki67 expression and Ki67 immunostaining in residual tumour

C-erbB2 expression was the only variable inversely associated with Ki67 reduction, whereas bcl-2 was the only directly associated independent variable (Table 2). Ki67 expression in residual tumour was directly associated with p53 at baseline, whereas pretreatment ER positivity was inversely associated (Table 2).

### Relapse-free survival according to clinical and biological variables

After a median follow-up of 52.7 months, 40 patients (25.5%) progressed and 25 (15.9%) died of disease. Patients attaining a complete clinical response showed a significantly longer RFS than those showing partial or no response ($P < 0.05$). A non-significant increased risk of recurrence was seen among patients with elevated Ki67 at baseline (Figure 3A). Conversely, a significant difference in RFS was observed when Ki67 expression at residual tumours was considered (Figure 3B).

When clinical response to treatment, menopausal status, histology grade, c-erbB2, bcl2, p53, steroid hormone receptor status, treatment administered, chemotherapy-induced Ki67 reduction, tumour dimension and lymph node status (evaluated clinically before treatment and pathologically afterwards) were considered together in a multivariate regression Cox model, clinical response ($P < 0.03$), tumour size ($P < 0.03$) and progesterone receptor status ($P < 0.02$) were independent predictors for disease recurrence, whereas postoperative lymph node status just failed to enter the model ($P = 0.07$).

### DISCUSSION

In this single institution experience involving a relatively large number of patients with operable breast cancer, our finding that chemotherapy is able to induce a reduction in the number of cells in the cell cycle confirms previous data (Daidone et al, 1991; Gardin et al, 1994; Frassoldati et al, 1997; Chang et al, 1999). Chemotherapy-induced reduction in Ki67 expression was found to be significantly associated with tumour shrinkage by both univariate and multivariate analysis. The latter finding is consistent with the notion that the antiproliferative effect of the cytotoxic agents is a mechanism of antitumour activity (Daidone et al, 1991; Change et al, 1999).

The patients in the present study were consecutively submitted to 2 different treatments, which led to a slight difference in the response rate. This may represent a limitation of our study. Another limitation is that tamoxifen has been found to have antiproliferative activity (Osborne et al, 1983; Clarke et al, 1993). As tamoxifen was added to chemotherapy in the first 76 cases, it is impossible in our study to ascertain the contribution the drug may have had in reducing the Ki67 expression. In order to avoid possible biases, the treatment was included among the independent variables in the multivariate analyses.

 *© 2001 Cancer Research Campaign*

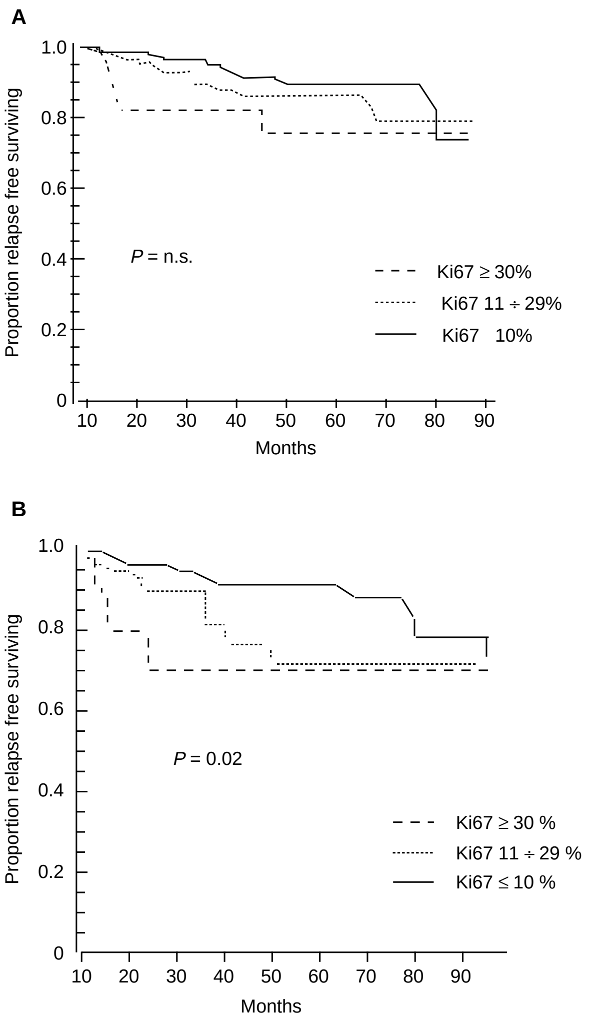

**Figure 3** Relapse free survival as a function of Ki67 expression at baseline (**A**) or at residual tumors after primary chemotherapy (**B**)

Two baseline markers, bcl2 and c-erbB2, in multivariate analysis were directly and inversely associated with a reduction in Ki67 expression, respectively. It has been shown that bcl2 is a marker of tumour responsiveness to endocrine therapy (Elledge et al, 1997). Therefore, the association of tamoxifen with chemotherapy in a number of patients may have contributed to the relationship between the reduction in Ki67 expression and pretreatment bcl2 immunostaining. The independent negative relationship between c-erbB2 expression and reduction in Ki67 suggests a blockade of the antiproliferative effect of chemo-endocrine therapy in c-erbB2-positive tumours. However, a possible concomitant antiapoptotic effect of c-erbB2 expression (Hamilton and Piccart, 2000) could not be excluded by our data.

Pretreatment Ki67 immunostaining showed a moderate but significant correlation with the antigen expression at mastectomy (or quadrantectomy). In other words, patients with rapidly proliferating BC before chemotherapy had a greater chance of maintaining elevated cell kinetic activity after treatment. In this sense, 2 baseline biological parameters, ER and p53, which correlated with low and elevated pre-chemotherapy Ki67 staining respectively, were also associated with Ki67 expression at the residual tumour. The relationship between Ki67 expression before and after treatment and the scarce influence of chemotherapy on p53, bcl2 and steroid hormone receptor status suggest that cytotoxic treatment reduces tumour size but has a limited effect on intrinsic tumour aggressiveness. Our findings are also consistent with the general concept that tumours with elevated proliferative activity usually have a poor prognosis in spite of a similar or even better response to the treatment administered (Gardin et al, 1994; Silvestrini et al, 1995; Bonetti et al, 1996).

Ki67 expression at first biopsy showed only a weak, inverse correlation with RFS; conversely, this inverse relationship attained statistical significance when Ki67 was evaluated at the end of treatment.

The implication is that the residual tumour is resistant to chemotherapy and that the proliferative activity assessed at this time has a greater value in predicting disease recurrence compared with baseline assessment. This observation may have direct clinical implications, because elevated proliferative activity in residual tumour suggests the persistence of aggressive disease and the need for subsequent adjuvant treatment possibly with non-cross-resistant schemes.

Patients whose breast cancers over-expressed c-erbB2 had similar response rates to either CMF + TAM or epirubicin, unlike the patients whose primary malignancy did not over-express c-erbB2. The latter finding seems to contrast with the data from the literature which indicate a role of c-erbB2 status in predicting the efficacy of both CMF and tamoxifen. Most of the published data refer to CMF or tamoxifen administered alone (Pegram et al, 1998; Hamilton and Piccart, 2000), but few data are available on the association of the 2 drugs. A comparative study in an adjuvant setting showed that c-erbB2 expression retained prognostic significance in the subgroups treated with CMF or tamoxifen but not in the group that received both drugs (Tetu and Brisson, 1994). Our results are consistent with this study. Even so, we cannot exclude the possibility of a type II error due to the small number of cases submitted to CMF + TAM.

In our series, clinical response was associated with a reduction in Ki67 expression, but changes in p53, c-erbB2, bcl2 and steroid hormone receptor status did not contribute to the treatment activity. It should be noted that the changes in Ki67 expression seen in the present study are relatively small, so that the treatment-induced tumour shrinkage is to be ascribed to either reduced proliferative activity and/or to increased apoptosis. The relationship between clinical response and reduction in Ki67 expression was already found in a previous neoadjuvant study with the chemo-endocrine regimen of mitoxantrone, methotrexate, mitomycin and tamoxifen (Chang et al, 1999). In contrast with our results, in this study clinical response, defined as complete response or minimal residual disease, was also associated with an increase of both PgR and bcl2 expression. Differences in cytotoxic drug administration, percent of patients who received tamoxifen (less than 50% in our study vs 100% in the Chang et al study) and response criteria adopted may account for the discrepancies observed.

One of the possible advantages in administering primary chemotherapy over adjuvant chemotherapy is that it allows the identification of surrogate parameters of treatment efficacy (Fisher and Mamounas, 1995). In our study the clinical response was significantly correlated with greater disease-free survival, thus confirming previous experiences (Bonadonna et al, 1998; Fisher et al, 1998). This correlation suggests that breast tumour response might be used as an indicator of the response of micrometastases

© 2001 Cancer Research Campaign 

to the therapy; however, this use needs to be formally validated in randomised clinical trials.

When multivariate survival analysis was performed to search for independent variables predicting for disease recurrence, tumour response but not Ki67 changes entered the Cox model. As previously discussed, reduction in kinetic cell activity is one way to achieve tumour shrinkage. For this reason it fails as an independent predictor of RFS when considered together with tumour response.

In conclusion, the administration of primary chemotherapy to breast cancer patients could offer clinical and biological advantages over adjuvant chemotherapy. It allows the clinician to better understand the mechanisms of the treatment activity and may provide surrogate parameters of treatment efficacy (Fisher et al, 1998). In this respect, we showed that chemo-endocrine therapy is able to induce tumour shrinkage and reduction in Ki67-stained cells, but its effect on tumour biology/aggressiveness is minimal. Reduction in the number of cells in the cell cycle correlates with tumour response but fails to be an independent predictor for RFS. This suggests that it is a mechanism of disease response. Ki67 expression after treatment was found to be a better predictor for disease relapse than the antigen expression at baseline.

### ACKNOWLEDGEMENTS

The authors wish to thank their nurses: Monia Balzani, Oriana Cervi, Francesca Ronchi and Nicoletta Zilioli for their co-operation. Supported in part by the Association: ‘Amici dell’ Ospedale di Cremona’ and a grant from the Consiglio Nazionale Ricerche (CNR), Rome, Italy.

### REFERENCES

Bonadonna G, Veronesi U, Brambilla C, Ferrari L, Luini A, Greco M, Bartoli C, Coopmans de Yoldi, Zucali R, Rilke F, Andreola S, Silvestrini R, Di Fronzo G and Valagussa P (1990) Primary chemotherapy to avoid mastectomy in tumours with diameters of three centimeters or more. *J Natl Cancer Inst* **82**: 1539–1545

Bonadonna G, Valagussa P, Brambilla C, Ferrari L, Molinterni A, Terenziani M and Zambetti M (1998) Primary chemotherapy in operable breast cancer: eight-years experience at the Milan Cancer Institute. *J Clin Oncol* **16**: 93–100

Bonetti A, Zaninelli M, Rodella S, Molino A, Sperotto L, Piubello Q, Bonetti F, Nortilli R, Turazza M and Cetto GL (1996) Tumor proliferative activity and response to first-line chemotherapy in advanced breast carcinoma. *Breast Cancer Res Treat* **38**: 289–297

Bottini A, Berruti A, Bersiga A, Brizzi MP, Brunelli A, Gorzegno G, DiMarco B, Aguggini S, Bolsi G, Cirillo F, Filippini L, Betri E, Bertoli G, Alquati P and Dogliotti L (2000) p53 but not bcl2 immunostaining is predictive of poor clinical complete response to primary chemotherapy in breast cancer patients. *Clin Cancer Res* **6**: 2751–2758

Chang J, Powles TJ, Allred DC, Ashley SE, Clark GM, Makris A, Assersohn L, Gregory RK, Osborne CK and Dowsett M (1999) Biologic markers as predictors of clinical outcome from systemic therapy for primary operable breast cancer. *J Clin Oncol* **17**: 3058–3063

Clarke RB, Laidlaw IJ, Jones LJ, Howell A and Anderson E (1993) Effect of tamoxifen in Ki67 labeling index in human breast tumors and its relationship to oestrogen and progesterone receptor status. *Br J Cancer* **67**: 606–611

Daidone MG, Silvestrini R, Valentinis B, Ferrari L and Bartoli C (1991) Changes in cell kinetics induced by primary chemotherapy in breast cancer. *Int J Cancer* **47**: 380–383

Dickson RB and Lippman ME (1996) Oncogenes and suppressor genes. In Harris JR, Lippman ME, Morrow M and Hellman S (eds): Diseases of the Breast. Philadelphia, Lippincott-Raven, pp 221–234

Elledge RM, Green S, Howes L, Clark GM, Berardo M, Allred DC, Pugh R, Ciocca D, Ravdin P, O’Sallivan J, Rivkin S, Martino S and Osborne CK (1997) Bcl-2, p53, and response to tamoxifen in estrogen receptor-positive metastatic breast cancer: a southwest oncology group study. *J Clin Oncol* **15**: 1916–1922

Elston CW and Ellis IO (1991) Pathological prognostic factors in breast cancer. I. The value of histological grade in breast cancer; experience from a large study with long-term follow-up. *Histopathology* **19**: 403–410

Fisher B and Mamounas EP (1995) Preoperative chemotherapy: a model for studying the biology and therapy of primary breast cancer. *J Clin Oncol* **13**: 537–540

Fisher B, Brown A, Mamounas E, Wieand S, Robidoux A, Margolese RG, Cruz AB, Jr., Fisher ER, Wickerham DL, Wolmark N, DeCillis A, Hoehn JL, Lees AW and Dimitrov NV (1997) The effect of preoperative therapy on local-regional disease in women with operable breast cancer: Findings from NSABP B-18. *J Clin Oncol* **15**: 2483–2493

Fisher B, Bryant J, Wolmark N, Mamounas E, Brown A, Fisher ER, Wickerham DL, Begovic M, DeCillis A, Robidoux A, Margolese RG, Cruz AB, Jr., Hoehn JL, Lees AW, Dimitrov NV and Bear HD (1998) Effect of preoperative chemotherapy on the outcome of women with operable breast cancer. *J Clin Oncol* **16**: 2672–2685

Frassoldati A, Adami F, Banzi C, Criscuolo M, Piccinini L and Silingardi V (1997) Changes of biological features in breast cancer cells determined by primary chemotherapy. *Breast Cancer Res Treat* **44(3)**: 185–192

Gardin G, Alama A, Rosso R, Rosso R, Campora E, Repetto L, Pronzato P, Merlini L, Naso C, Camoriano A, Meazza R, Barbieri F, Baldini E, Giannessi PG and Conte PF (1994) Relationship of variations in tumor cell kinetics induced by primary chemotherapy to tumor regression and prognosis in locally advanced breast cancer. *Breast Cancer Res Treat* **32**: 311–318

Hamilton A and Piccart M (2000) The contribution of molecular markers to the prediction of response in the treatment of breast cancer: A review of the literature on HER-2, p53 and bcl-2. *Ann Oncol* **11(6)**: 647–663

Makris A, Powels TJ, Ashley SE, Chang J, Hickish T, Tidy VA, Nash AG and Ford HT (1998). A reduction in the requirement for mastectomy in a randomized trial of neoadjuvant chemoendocrine therapy in primary breast cancer. *Ann Oncol* **9**: 1179–1184

Mauriac L, Durand M, Avril A and Dihuydy JM (1991) Effects of primary chemotherapy in conservative treatment of breast cancer patients with operable tumours larger than 3 cm. *Ann Oncol* **2**: 347–354

McCarty KS, Jr., Miller LS, Cox EB, Konrath J and McCarty KS, Sr. (1985) Estrogen receptor analyses. *Arch Pathol Lab Med* **109**: 716–721

Osborne CK, Boldt DH, Clark GM and Trent JM (1983) Effects of tamoxifen on human breast cancer cell cycle kinetics: accumulation of cells in early G1 phase. *Cancer Res* **43(8)**: 3583–3585

Pegram MD, Pauletti G and Slamon DJ (1998) Her-2/neu as a predictive marker of response to breast cancer therapy. *Breast Cancer Res Treat* **52**: 65–77

Powles TJ, Hickish TF, Makris A, Ashley SE, O’Brien ME, Tidy VA, Casey S, Nash AG, Sacks N and Cosgrove D (1995) Randomized trial of chemoendocrine therapy started before or after surgery for treatment of primary breast cancer. *J Clin Oncol* **13**: 547–552

Robertson JFR, Ellis IO, Elston CV and Blamey RW (1992) Mastectomy or tamoxifen as initial therapy for operable breast cancer: 5-years follow-up. *Eur J Cancer* **28**: 908–910

Silvestrini R, Benini E, Daidone MG, Veneroni S, Boracchi P, Cappelletti V, Di Fronzo G and Veronesi U (1993) p53 as an independent prognostic marker in lymph node-negative breast cancer patients. *J Natl Cancer Inst* **85**: 965–970

Silvestrini R, Daidone MG, Luisi A, Boracchi P, Mezzetti M, Di Fronzo G, Andreola S, Salvadori B and Veronesi U (1995) Biologic and clinicopathologic factors as indicators of specific relapse types in node-negative breast cancer. *J Clin Oncol* **13**: 697–704

Statsoft Inc. (1995) Statistica for Windows, Rel. 5.0. 2325 East 13th Street, Tulsa OK 74104

Tetu B, and Brisson J (1994) Prognostic significance of HER-2/neu oncoprotein expression in node-positive breast cancer. The influence of the pattern of immunostaining and adjuvant therapy. *Cancer* **73**: 2359–2365

World Health Organization (1978) WHO handbook for reporting results of cancer treatment. WHO offset publication. Geneva, Switzerland, UICC

 *© 2001 Cancer Research Campaign*
